# Supplementary material for: High-Resolution Melting assays development for discrimination of fungal pathogens causing Grapevine Trunk Diseases
Source: PLoS One. 2025 Dec 1;20(12):e0331101. doi: 10.1371/journal.pone.0331101 (PMC12668526; doi:10.1371/journal.pone.0331101)
Supplement: S1 File — (DOCX) [file pone.0331101.s001.docx]

| Isolate | Fungal Species | Accession ITS | Accession Tub2 |
| --- | --- | --- | --- |
| 100.1 | *N. parvum* | PQ556103.1 | PQ566272.1 |
| 143.1 | *N. parvum* | PQ556106.1 | PQ566275.1 |
| 98.1 | *N. parvum* | PQ556125.1 | PQ566294.1 |
| 160.2 | *N. luteum* | PQ556076.1 | PQ566245.1 |
| 34.1 | *N. luteum* | PQ556072.1 | PQ566241.1 |
| 35.1 | *N. luteum* | PQ556073.1 | PQ566242.1 |
| 94.1 | *B. dothidea* | PQ555936.1 | PQ566106.1 |
| 96.1 | *B. dothidea* | PQ555937.1 | PQ566107.1 |
| 100.3 | *B. dothidea* | PQ555938.1 | PQ566108.1 |
| 16.1 | *Da. seriata* | PQ555958.1 | PQ566128.1 |
| 21.5 | *Da. seriata* | PQ556022.1 | PQ566192.1 |
| 124.1 | *Da. seriata* | PQ556031.1 | PQ566201.1 |
| 107.1 | *Da. mutila* | PQ555951.1 | PQ566121.1 |
| 98.3 | *E. lata* | PQ555862.1 | PQ566044.1 |
| 46.3 | *E. lata* | PQ555860.1 | PQ566042.1 |
| 49.8 | *E. lata* | PQ555861.1 | PQ566043.1 |
| 39.1 | *Di. ampelina* | PQ555880.1 | PQ566053.1 |
| 153.1 | *Di. ampelina* | PQ555898.1 | PQ566071.1 |
| 67.2 | *Di. ampelina* | PQ555887.1 | PQ566060.1 |
| 20.2 | *Pm. minimum* | PQ555849.1 | PQ566031.1 |
| 206.7 | *Pm. minimum* | PQ556103.1 | PQ566032.1 |
| 20.1 | *Pm. minimum* | PQ556106.1 | PQ566039.1 |
| 66.1 | *Pa. chlamydospora* | PQ555755.1 |  |
| 66.2 | *Pa. chlamydospora* | PQ555822.1 |  |
| 293.2 | *Pa. chlamydospora* | PQ555754.1 |  |
| 295.1 | *F. mediterranea* | PQ556125.1 |  |
| 36.2 | *F. mediterranea* | PQ556076.1 |  |
| 23.2 | *F. mediterranea* | PQ556072.1 |  |
