# Supplementary material for: High-Resolution Melting assays development for discrimination of fungal pathogens causing Grapevine Trunk Diseases
Source: PLoS One. 2025 Dec 1;20(12):e0331101. doi: 10.1371/journal.pone.0331101 (PMC12668526; doi:10.1371/journal.pone.0331101)
Supplement: S2 File — (DOCX) [file pone.0331101.s002.docx]

| *Diaporthe ampelina* | *Eutypa lata* | *Phaeomoniella chlamydospora* | *Phaeoacremonium minimum* | *Fomitiporia mediterranea* | *Botryosphaeria dothidea* | *Diplodia mutila* | *Diplodia seriata* | *Neofusicoccum luteum* | *Neofusicoccum parvum* |
| --- | --- | --- | --- | --- | --- | --- | --- | --- | --- |
| ON082776.1 | OP079829.1 | OP079854.1 | ON238015.1 | XM_007269132.1 | OM801236.1 | ON082775.1 | ON098140.1 | OL901284.1 | OM974143.1 |
| OP663196.1 | OP079831.1 | OP079856.1 | ON238016.1 | XM_007269133.1 | OM822674.1 | OL901282.1 | OM836559.1 | OL901287.1 | OM801233.1 |
| OP663197.1 | OP079832.1 | OP079888.1 | ON238017.1 |  | OM468557.1 | OL901297.1 | OL901296.1 | OL901295.1 | OM801237.1 |
| OP663198.1 | OP079833.1 | OP079892.1 | ON238018.1 |  | OM262439.1 | OL901300.1 | OL677455.1 | OL901301.1 | ON082772.1 |
| OP663199.1 | OP079834.1 | OP079895.1 | ON238019.1 |  | OM262440.1 | OL901309.1 | OP428705.1 | OL901305.1 | ON098141.1 |
| OP663200.1 | OP079842.1 | OP079896.1 | ON887279.1 |  | OM262441.1 | OL901318.1 | OP079865.1 | OL901308.1 | ON098143.1 |
| OP663201.1 | OP079843.1 | OP079899.1 | OP079855.1 |  | OM262442.1 | OL901327.1 | OP079866.1 | OL901310.1 | OP807950.1 |
| OP663202.1 | MZ476772.1 | MK070488.1 | OP079857.1 |  | OM262443.1 | OL901339.1 | OP079902.1 | OL901312.1 | OM262433.1 |
| OP663203.1 | OK287408.1 | MK070489.1 | OP079861.1 |  | OM262444.1 | OL901341.1 | MZ418112.1 | OL901313.1 | OM262434.1 |
| OP663204.1 | MZ277248.1 | MK070490.1 | OP079862.1 |  | OL901288.1 | OL901363.1 | MZ418113.1 | OL901314.1 | OM262435.1 |
| OP663205.1 | MZ277249.1 | MN245021.1 | OP079889.1 |  | OL901289.1 | OL901368.1 | MW596890.1 | OL901315.1 | OM262436.1 |
| OP663206.1 | MZ277250.1 | MK903786.1 | OP079890.1 |  | OL901290.1 | OP373140.1 | OM063099.1 | OL901323.1 | OM262437.1 |
| OP663207.1 | MZ277251.1 | MK903787.1 | OP079893.1 |  | OL901291.1 | ON054940.1 | OL362020.1 | OL901326.1 | OM262438.1 |
| OP663208.1 | MZ277252.1 | MK903788.1 | OP079894.1 |  | OL901292.1 | ON054941.1 | OK489796.1 | OL901332.1 | OL442004.1 |
| OP663209.1 | MZ277253.1 | MK903789.1 | OP079897.1 |  | OL901293.1 | ON054939.1 | OK587398.1 | OL901335.1 | OL442005.1 |
| OP663210.1 | MW366817.1 | MK903790.1 | OP079898.1 |  | OL901294.1 | OM676658.1 | OK587400.1 | OL901343.1 | OL442006.1 |
| OP079904.1 | MW366818.1 | MK903791.1 | OP079900.1 |  | OL901298.1 | MW596891.1 | OK571383.1 | OL901347.1 | OL442007.1 |
| OP079905.1 | MW366819.1 | MK903792.1 | OP079901.1 |  | OL901299.1 | MZ542354.1 | OK346634.1 | OL901349.1 | OL442008.1 |
| OP079906.1 | MW366820.1 | MK903793.1 | MW856465.1 |  | OL901304.1 | MZ073930.1 | OK346635.1 | OL901350.1 | OL442009.1 |
| OP079908.1 | MN839600.1 | MK903794.1 | MZ442471.1 |  | OL901307.1 | MZ073931.1 | OK489792.1 | OL901351.1 | OL442010.1 |
| MT880113.1 | MN245030.1 | MK903795.1 | MZ442472.1 |  | OL901316.1 | MZ073932.1 | OK489794.1 | OL901352.1 | OL442011.1 |
| MT880114.1 | MN433693.1 | MK903796.1 | MZ442473.1 |  | OL901317.1 | MT813196.1 | OK571380.1 | OL901354.1 | OL442012.1 |
| MT880115.1 | MN433695.1 | MK903797.1 | MT598111.1 |  | OL901320.1 | MW574060.1 | OK571381.1 | OL901356.1 | OL442013.1 |
| MZ555778.1 | MN329778.1 | MK903798.1 | MT598112.1 |  | OL901325.1 | MW574061.1 | OK571382.1 | OL901359.1 | OL901281.1 |
| MZ555779.1 | MN329779.1 | MK903799.1 | MT598113.1 |  | OL901328.1 | MW574062.1 | OK571384.1 | OL901362.1 | OL901285.1 |
| MZ555780.1 | MN329780.1 | MK903800.1 | MT598114.1 |  | OL901329.1 | MW574063.1 | MZ423122.1 | MZ476038.1 | OL901286.1 |
| MZ555781.1 | MN329781.1 | MK903801.1 | MT585645.1 |  | OL901331.1 | MW574064.1 | MZ423123.1 | MZ476039.1 | OL901302.1 |
| MZ555782.1 | MN078073.1 | MK903802.1 | MT585646.1 |  | OL901345.1 | MW574065.1 | MZ423124.1 | OM515109.1 | OL901306.1 |
| MZ555783.1 | MN078074.1 | MK903803.1 | MT585647.1 |  | OL901355.1 | MW419212.1 | MT914171.1 | OM515110.1 | OL901321.1 |
| MZ555784.1 | MN078075.1 | MK903804.1 | MT585648.1 |  | OL901366.1 | MT592509.1 | MT914174.1 | MW419253.1 | OL901322.1 |
| MZ555785.1 | MN078076.1 | MK903805.1 | MT585649.1 |  | ON887280.1 | MT592510.1 | MW884091.1 | MW419254.1 | OL901324.1 |
| MZ555786.1 | MN629335.1 | MK903806.1 | MT585650.1 |  | OK480599.1 | MT592511.1 | MT813195.1 | MT592687.1 | OL901330.1 |
| MZ555787.1 | MN629336.1 | MK903807.1 | MT585651.1 |  | OK480600.1 | MT592512.1 | MW574053.1 | MT592688.1 | OL901337.1 |
| MZ555788.1 | MN629337.1 | MK903808.1 | MT585652.1 |  | OM720127.1 | MT592513.1 | MW574054.1 | MT592689.1 | OL901353.1 |
| MZ555789.1 | MH925329.1 | MK903809.1 | MT585653.1 |  | ON191051.1 | MT592514.1 | MW574055.1 | MT592690.1 | OL901357.1 |
| MZ555790.1 | MH925330.1 | MK903810.1 | MT585654.1 |  | ON191052.1 | MT592515.1 | MW574056.1 | MT592691.1 | OL901358.1 |
| MZ555791.1 | MG745801.1 | MK903811.1 | MT585655.1 |  | ON191053.1 | MT044309.1 | MW574057.1 | MT592692.1 | OL901360.1 |
| MZ555792.1 | MG745802.1 | MK903812.1 | MT585656.1 |  | ON191054.1 | MT044310.1 | MW574058.1 | MT592693.1 | OL901361.1 |
| MZ555793.1 | MG366118.1 | MK903813.1 | MT585657.1 |  | ON191055.1 | MT309386.1 | MW574059.1 | MT592694.1 | OL901367.1 |
| MZ555794.1 | MG366119.1 | MK903814.1 | MT585658.1 |  | ON191056.1 | MN318126.1 | MW419230.1 | MT592695.1 | OL901369.1 |
| MZ555795.1 | MG978306.1 | MK903815.1 | MT585659.1 |  | ON191057.1 | MN318127.1 | MW419231.1 | MT592696.1 | OL901370.1 |
| MZ555796.1 | MG978307.1 | MK903816.1 | MT585660.1 |  | ON191058.1 | MN318128.1 | MW419232.1 | MT164530.1 | OL901372.1 |
| MZ555797.1 | MG978308.1 | MK903817.1 | MT585661.1 |  | ON191059.1 | MN318129.1 | MW419233.1 | MT309397.1 | OL790430.1 |
| MZ555798.1 | MG978309.1 | MK903818.1 | MT585662.1 |  | ON191060.1 | MG952716.1 | MW419234.1 | MH118938.1 | OP561950.1 |
| MZ555799.1 | MF359689.1 | MK903819.1 | MT585663.1 |  | ON191061.1 | MG952717.1 | MW419235.1 | MH006966.1 | OP561952.1 |
| MZ555800.1 | MF359690.1 | MK903820.1 | MT585664.1 |  | ON191062.1 | MG952718.1 | MW419236.1 | MH006967.1 | MZ546153.1 |
| MZ555801.1 | KY752793.1 | MK903821.1 | MT294093.1 |  | ON191063.1 | MG952719.1 | MW419237.1 | MH006968.1 | MZ546154.1 |
| MZ555802.1 | KY752794.1 | MK903822.1 | MT294094.1 |  | ON191064.1 | MH922944.1 | MW419238.1 | KY000176.1 | MZ546155.1 |
| MZ555803.1 | KY752795.1 | MK903823.1 | MT294095.1 |  | ON191065.1 | MH922945.1 | MT592533.1 | KY000178.1 | ON933625.1 |
| MZ555804.1 | KY752796.1 | MK903824.1 | MT294096.1 |  | ON191066.1 | MH922946.1 | MT592534.1 | KY000183.1 | ON933626.1 |
| MZ555805.1 | KY752797.1 | MK903825.1 | MT294097.1 |  | ON191067.1 | MH057752.1 | MT592535.1 | KY000184.1 | ON933627.1 |
| MZ555806.1 | KY111613.1 | MK903826.1 | MT294098.1 |  | ON191068.1 | MG745799.1 | MT592536.1 | KY000142.1 | ON933628.1 |
| MZ634295.1 | KY111614.1 | MK903827.1 | MT294099.1 |  | ON191069.1 | MH118936.1 | MT592537.1 | KY000174.1 | OM397539.1 |
| MZ634296.1 | KY111615.1 | MK903828.1 | MT294100.1 |  | ON191070.1 | MG015815.1 | MT592538.1 | KY000175.1 | OM397540.1 |
| MZ634297.1 | KY111616.1 | MK903829.1 | MT032151.1 |  | ON191071.1 | MG015816.1 | MT592539.1 | KY000177.1 | LC722361.1 |
| MZ634298.1 | KY111617.1 | MK903830.1 | MT032152.1 |  | ON191072.1 | KY644558.1 | MT592540.1 | KY000179.1 | LC722363.1 |
| MZ634299.1 | KM851439.1 | MK903831.1 | MT032153.1 |  | ON191073.1 | KY644560.1 | MT592541.1 | KY000185.1 | OL694623.1 |
| MZ634300.1 | KM851440.1 | MK903832.1 | MT032154.1 |  | ON191074.1 | KY644559.1 | MT592542.1 | KY000200.1 | ON461897.1 |
| MZ634301.1 | KM851441.1 | MK903833.1 | MT032157.1 |  | ON191075.1 | KY393215.1 | MT592543.1 | KY000204.1 | MZ439862.1 |
| MZ634302.1 | KM851442.1 | MK903834.1 | MN839602.1 |  | ON191076.1 | KY393217.1 | MT592544.1 | KY000215.1 | OL455946.1 |
| MZ634303.1 | KM851443.1 | MK903835.1 | MN634158.1 |  | ON191077.1 | KY554743.1 | MT592545.1 | KY000217.1 | OL455947.1 |
| MT903966.1 | KM851444.1 | MK903836.1 | MN634159.1 |  | OK337616.1 | KY554744.1 | MT592546.1 | KY436375.1 | ON041445.1 |
| MT903967.1 | KM851445.1 | MK903837.1 | MN634160.1 |  | MW853968.1 | KX901801.1 | MT592547.1 | KX871754.1 | ON000394.1 |
| MT903968.1 | KM851446.1 | MK903838.1 | MN634161.1 |  | MW853969.1 | KX901802.1 | MT592548.1 | KX871755.1 | ON000395.1 |
| MK838570.1 | KM851447.1 | MK903839.1 | MN634162.1 |  | MW853970.1 | KX901803.1 | MT592549.1 | KX871756.1 | MZ564201.1 |
| MK838571.1 | KM851448.1 | MK903840.1 | MN634163.1 |  | MW864163.1 | KX011910.1 | MT592550.1 | KX871757.1 | MZ564202.1 |
| MG812588.1 | KM851449.1 | MK903841.1 | MN634164.1 |  | MW864164.1 | KU065142.1 | MT592551.1 | KX871758.1 | MZ564203.1 |
| MG812589.1 | KM851450.1 | MN747131.1 | MN634165.1 |  | MW864165.1 | KP699092.1 | MT592552.1 | KX871759.1 | MZ564207.1 |
| KY511341.1 | KU376435.1 | MN747132.1 | MN634166.1 |  | MW864166.1 | KU198426.1 | MT592553.1 | KX871760.1 | MZ564209.1 |
| KY511342.1 | JX978578.1 | MG745809.1 | MN634167.1 |  | MW818442.1 | KT954167.1 | MT592554.1 | KX871761.1 | MZ564213.1 |
| KY511343.1 | JX978579.1 | MG745810.1 | MN634168.1 |  | MW818443.1 | KP762477.1 | MT592555.1 | KX871762.1 | MZ564216.1 |
| KY511353.1 | JX978580.1 | MG745811.1 | MN634169.1 |  | OK136105.1 | KP762478.1 | MT592556.1 | KX871763.1 | MZ564234.1 |
| KY511354.1 | JX978581.1 | KP721669.1 | MK759833.1 |  | OK136106.1 | KP762479.1 | MT592557.1 | KX464956.1 | MZ564235.1 |
| MK183121.1 | JX978582.1 | KP721670.1 | MK759837.1 |  | OM515102.1 | KP762480.1 | MT592558.1 | KX464966.1 | MZ564236.1 |
| MH051284.1 | JX978583.1 | KP721671.1 | MT122930.1 |  | OM515103.1 | KP762481.1 | MT592559.1 | KX464967.1 | MZ564237.1 |
| MG281143.1 | KF453525.1 | KP721672.1 | MT122931.1 |  | OM515104.1 | KF778884.1 | MT592560.1 | KX464968.1 | MZ564239.1 |
| MG281144.1 | KF453526.1 | KP721673.1 | MT122932.1 |  | OM515105.1 | KF778885.1 | MT592561.1 | KX464969.1 | MZ564241.1 |
| MG281145.1 | KF453527.1 | KR260465.1 | MT122933.1 |  | OM515107.1 | KF778886.1 | MT592562.1 | KX464970.1 | MZ564244.1 |
| MG281146.1 | KF453528.1 | KP213108.1 | MT122934.1 |  | OM515108.1 | KF778887.1 | MT592563.1 | KX505929.1 | MZ564246.1 |
| MG281147.1 | KF453529.1 | KF764660.1 | MT122935.1 |  | OM515106.1 | KF778888.1 | MT592564.1 | KX453297.1 | MZ564247.1 |
| MG281148.1 | KF453530.1 | KF764661.1 | MT122936.1 |  | MZ363819.1 | KF778889.1 | MT592565.1 | KP860768.1 | MZ564249.1 |
| MG281149.1 | KF453531.1 | KF764662.1 | MT122937.1 |  | ON185541.1 | KF575097.1 | MT592566.1 | KP860782.1 | MZ564250.1 |
| MG281150.1 | JX515680.1 | KF764663.1 | MN166033.1 |  | OL827572.1 | KF575098.1 | MT592567.1 | KP860783.1 | MZ564252.1 |
| MG281151.1 | JX515681.1 | KF764664.1 | MN166034.1 |  | OK669147.1 | KF575099.1 | MT592568.1 | KP860787.1 | MZ564254.1 |
| MG281152.1 | JX515682.1 | KF764665.1 | MN166035.1 |  | OM328347.1 | KC480173.1 | MT592569.1 | KP860789.1 | MZ564255.1 |
| MG281153.1 | JX515683.1 | KF764666.1 | MN166036.1 |  | OM328345.1 | JX515674.1 | MT592570.1 | JX515686.1 | MZ564256.1 |
| MG281154.1 | JN975375.1 | KF764667.1 | MN166037.1 |  | OM328346.1 | JX515675.1 | MT592571.1 | JX515687.1 | MZ418115.1 |
| MG281155.1 | JN975376.1 | KF764668.1 | MN166038.1 |  | OL891743.1 | JX431880.1 | MT592572.1 | JX898979.1 | MZ418107.1 |
| MG281156.1 | JN975377.1 | KF764683.1 | MN166039.1 |  | OL891744.1 | JX089969.1 | MT592573.1 | JX898980.1 | MZ418109.1 |
| MG281157.1 | JN975378.1 | JX515697.1 | MN166040.1 |  | OL891745.1 | JQ411442.1 | MT592574.1 | JX898981.1 | MZ418116.1 |
| MG281158.1 | JN975379.1 | JX515698.1 | MN166041.1 |  | OL891746.1 | JQ411443.1 | MT063125.1 | JX898982.1 | MZ418117.1 |
| MG281159.1 | JN975380.1 | JX679869.1 | MN166042.1 |  | OL891747.1 | JQ411444.1 | MT063126.1 | JX898983.1 | OM039456.1 |
| MG281160.1 | JN975381.1 | JX679877.1 | MN166043.1 |  | OL891748.1 | JQ411459.1 | MT063127.1 | JX898984.1 | OK337998.1 |
| MG281161.1 | JN975382.1 | JX679878.1 | MN166044.1 |  | OK484567.1 | JQ411460.1 | MT063128.1 | JX898985.1 | MW596892.1 |
| MG281162.1 | JN975383.1 | JX679879.1 | MK994191.1 |  | MZ934655.1 | JQ659295.1 | MT063129.1 | JX898986.1 | OM453641.1 |
| MG281163.1 | JN975384.1 | JX679880.1 | MK994192.1 |  | OK146905.1 | JQ659296.1 | MT063130.1 | JX898987.1 | OL891749.1 |
| MG281164.1 | JN975385.1 | JX679881.1 | MK994193.1 |  | OK146909.1 | JN595814.1 | MT063131.1 | JX089970.1 | OL891750.1 |
| MG281165.1 | JN975386.1 | JX679885.1 | MK875275.1 |  | MZ423131.1 | JN595820.1 | MT063132.1 | JQ823022.1 | OL891751.1 |
| MG281166.1 | JN975387.1 | JX679886.1 | MH729082.1 |  | MZ358258.1 | JN017908.1 | MT063133.1 | JQ080550.1 | OL891752.1 |
| MG281167.1 | JN975388.1 | JX679870.1 | MH729083.1 |  | MZ358259.1 | JF271769.1 | MT063134.1 | JN595817.1 | OL891753.1 |
| MG281168.1 | JN975389.1 | JX679871.1 | MH729084.1 |  | MZ358260.1 | GU251776.1 | MT063135.1 | JN595818.1 | OL891754.1 |
| MG281169.1 | JN975390.1 | JX679872.1 | MG745806.1 |  | MZ358261.1 | GU251777.1 | MT063136.1 | JN595822.1 | OL891755.1 |
| MG281170.1 | JN975391.1 | JX679873.1 | MG745807.1 |  | MZ358262.1 | GU251778.1 | MT063137.1 | HQ529773.1 | OL891756.1 |
| MG281171.1 | JN975392.1 | JX679874.1 | MG745808.1 |  | MZ358263.1 | GU251779.1 | MT063138.1 | HQ529726.1 | OL891757.1 |
| MG281172.1 | JN975393.1 | JX679875.1 | MG456738.1 |  | MT874970.1 | DQ458849.1 | MT063139.1 | HQ529727.1 | OL891758.1 |
| MG281173.1 | JN975394.1 | JX679876.1 | MF489025.1 |  | MT874971.1 | DQ458850.1 | MT063140.1 | HQ529728.1 | OL891759.1 |
| KY923779.1 | JN975395.1 | JX679882.1 | MF489026.1 |  | MT874972.1 | DQ458851.1 | MT063141.1 | HQ529729.1 | OL891760.1 |
| KY923780.1 | JN975396.1 | JX679883.1 | MF489027.1 |  | MW884086.1 | DQ458852.1 | MT063142.1 | HQ529730.1 | OL955494.1 |
| KY923781.1 | HQ692493.1 | JX679884.1 | MF489028.1 |  | MW884087.1 | DQ008336.1 | MT063143.1 | HQ529731.1 | OL955495.1 |
| KY923782.1 | HQ692494.1 | JX679887.1 | MF489029.1 |  | MZ848192.1 | DQ233619.1 | MT063144.1 | HQ529732.1 | OL955496.1 |
| KY923783.1 | HQ692495.1 | JX679888.1 | MF489030.1 |  | MT513138.1 | DQ233620.1 | MT063145.1 | HQ529733.1 | OL955497.1 |
| KY923784.1 | HQ692496.1 | JX679889.1 | MF489031.1 |  | MT513139.1 | AY972121.1 | MT063146.1 | JF921872.1 | OL955498.1 |
| KY923785.1 | HQ692497.1 | JX679890.1 | MF489032.1 |  | MT513140.1 | AY972122.1 | MT063147.1 | JF921873.1 | OL955499.1 |
| KY923786.1 | HQ692498.1 | HQ288313.1 | MF489033.1 |  | MT513141.1 | AY236933.1 | MT063148.1 | JF921874.1 | OL955500.1 |
| KY923787.1 | HQ692499.1 | HQ288314.1 | MF489034.1 |  | MT513142.1 |  | MT063149.1 | JN017909.1 | MZ229675.1 |
| KY923788.1 | HQ692500.1 | HQ288315.1 | KY906739.1 |  | MZ416742.1 |  | MT063150.1 | JF271773.1 | MZ229676.1 |
| KY923789.1 | HQ692501.1 | HQ288316.1 | KY906757.1 |  | MT265235.1 |  | MT063151.1 | JF271781.1 | MZ229677.1 |
| KY923790.1 | HQ288299.1 | GQ903723.1 | KY906813.1 |  | MZ399162.1 |  | MT063152.1 | JF271782.1 | MZ229678.1 |
| KY923791.1 | HQ288300.1 | GQ903724.1 | KY906837.1 |  | MZ197998.1 |  | MT063153.1 | HQ392737.1 | MZ229679.1 |
| MF409159.1 | HM164768.1 | GQ903725.1 | KY906843.1 |  | MZ197999.1 |  | MT063154.1 | HQ392741.1 | OL589061.1 |
| KY887663.1 | HM164770.1 | EU078329.1 | KY906847.1 |  | MW051690.1 |  | MN600978.1 | HQ392746.1 | MZ423132.1 |
| KX256214.1 | HM164739.1 | EU078330.1 | KY906887.1 |  | MW051691.1 |  | MN600979.1 | HQ392747.1 | MT874975.1 |
| KP721683.1 | HM164740.1 | EU078331.1 | KY906895.1 |  | MW561785.1 |  | MN600980.1 | HQ392750.1 | MW790289.1 |
| KP721684.1 | HM164741.1 | EU078332.1 | KY906947.1 |  | MW561786.1 |  | MN600981.1 | HQ392751.1 | MW266970.1 |
| KP721685.1 | HM164742.1 | EU078333.1 | KY906949.1 |  | MW561787.1 |  | MN600982.1 | HQ392752.1 | MW266971.1 |
| KP721686.1 | HM164743.1 | EU078334.1 | MF352202.1 |  | MW561788.1 |  | MN245025.1 | HQ392761.1 | MW266972.1 |
| KP721687.1 | HM164744.1 | AF253968.1 | MF352203.1 |  | MW561789.1 |  | MN318124.1 | HQ392762.1 | MW266973.1 |
| KC849460.1 | HM164745.1 | AF253969.1 | KY312678.1 |  | MW561790.1 |  | MN318125.1 | GU251881.1 | MW266974.1 |
| KC343984.1 | HM164746.1 |  | KY312679.1 |  | MW561791.1 |  | MG952720.1 | GQ857664.1 | MW625871.1 |
| KC343985.1 | HM164747.1 |  | KY320265.1 |  | MW561792.1 |  | MG952721.1 | EU673092.1 | MT775857.1 |
| KC343986.1 | HM164748.1 |  | KY320266.1 |  | MW561793.1 |  | MG952722.1 | EU328264.1 | MW517567.1 |
| JX275452.1 | HM164749.1 |  | KY320267.1 |  | MW561794.1 |  | MN373271.1 | DQ458848.1 | MW517568.1 |
| HQ288317.1 | HM164750.1 |  | KY320268.1 |  | MW561795.1 |  | MK522086.1 | DQ233625.1 | MW517569.1 |
| HQ288318.1 | HM164751.1 |  | KY320269.1 |  | MW561796.1 |  | MK522087.1 | DQ233626.1 | MZ561053.1 |
| HQ288319.1 | HM164752.1 |  | KY320270.1 |  | MW561797.1 |  | MK522088.1 | DQ233627.1 | MT832776.1 |
| HQ288320.1 | HM164753.1 |  | KY320271.1 |  | MW561798.1 |  | MK733738.1 | DQ233628.1 | MT832777.1 |
| HQ288321.1 | HM164754.1 |  | KY012296.1 |  | MW561799.1 |  | MK318282.1 | DQ233629.1 | MT832778.1 |
| HQ586905.1 | HM164755.1 |  | KY012297.1 |  | MW561800.1 |  | MK281579.1 | DQ233630.1 | MT832779.1 |
| HQ586906.1 | HM164756.1 |  | KY012298.1 |  | MW561801.1 |  | MK388681.1 | AY339249.1 | MT832780.1 |
| HQ586909.1 | HM164757.1 |  | KY012299.1 |  | MW561802.1 |  | MK388682.1 | AY339250.1 | MT832781.1 |
| HQ586913.1 | HM164758.1 |  | KY012300.1 |  | MW561803.1 |  | MH745086.1 | AY339251.1 | MT832790.1 |
| HQ586915.1 | HM164759.1 |  | KY012301.1 |  | MW561804.1 |  | MH745087.1 | AY236922.1 | MT832791.1 |
| HQ586921.1 | HM164760.1 |  | KY012302.1 |  | MW561805.1 |  | MH745088.1 | AY236923.1 | MT832795.1 |
| HQ586922.1 | HM164761.1 |  | KY012303.1 |  | MW561806.1 |  | MH745089.1 |  | MT832798.1 |
| HQ586923.1 | HM164762.1 |  | KY012304.1 |  | MW561807.1 |  | MH745090.1 |  | MT832802.1 |
| HQ586925.1 | HM164763.1 |  | KY012305.1 |  | MW561808.1 |  | MH745091.1 |  | MT832809.1 |
| HQ586930.1 | HM164764.1 |  | KY012306.1 |  | MW561809.1 |  | MK033134.1 |  | MT832810.1 |
| HQ586931.1 | HM164765.1 |  | KY012307.1 |  | MW561810.1 |  | MH249049.1 |  | MT832811.1 |
| GU294718.1 | HM164766.1 |  | KY012308.1 |  | MW561811.1 |  | MG745800.1 |  | MT832812.1 |
| GU294719.1 | HM164767.1 |  | KY030790.1 |  | MW561812.1 |  | MH221110.1 |  | MT832815.1 |
| GU294720.1 | GQ294007.1 |  | KY030791.1 |  | MW561813.1 |  | MH221111.1 |  | MT832816.1 |
| GU294725.1 | DQ006965.1 |  | KU737515.1 |  | MW561814.1 |  | MH221114.1 |  | MT832817.1 |
|  | DQ006970.1 |  | KU737516.1 |  | MW561815.1 |  | MH221115.1 |  | MT832818.1 |
|  | DQ006964.1 |  | KU720063.1 |  | MW561816.1 |  | MH221118.1 |  | MT832820.1 |
|  | DQ006975.1 |  | KU720064.1 |  | MW561817.1 |  | MH221119.1 |  | MT832822.1 |
|  | DQ006969.1 |  | KU095817.1 |  | MW561818.1 |  | MH221104.1 |  | MT832823.1 |
|  | DQ006972.1 |  | KU094046.1 |  | MW561819.1 |  | MH221105.1 |  | MT832827.1 |
|  | DQ006973.1 |  | KU094047.1 |  | MW561820.1 |  | MH221106.1 |  | MZ153111.1 |
|  | DQ006985.1 |  | KP721674.1 |  | MW561821.1 |  | MH221107.1 |  | MT265233.1 |
|  | DQ006986.1 |  | KP721675.1 |  | MW561822.1 |  | MH118937.1 |  | MZ398260.1 |
|  | DQ006987.1 |  | KP721676.1 |  | MW561823.1 |  | MG548238.1 |  | MW435151.1 |
|  | DQ006988.1 |  | KP721677.1 |  | MW561824.1 |  | MG548239.1 |  | MW435152.1 |
|  | DQ006989.1 |  | KP721678.1 |  | MW561825.1 |  | MG548240.1 |  | MW435153.1 |
|  | DQ006990.1 |  | KP721679.1 |  | MW561826.1 |  | MG548241.1 |  | OU022063.1 |
|  | DQ006991.1 |  | KP721680.1 |  | MW561827.1 |  | MG548242.1 |  | OU022064.1 |
|  | DQ006992.1 |  | KP721681.1 |  | MW561828.1 |  | MG548243.1 |  | OU022065.1 |
|  | DQ006993.1 |  | KP721682.1 |  | MW561829.1 |  | MG548244.1 |  | OU022066.1 |
|  | DQ006994.1 |  | KR003968.1 |  | MW561830.1 |  | MG548245.1 |  | OU022067.1 |
|  | DQ006995.1 |  | KR003969.1 |  | MW561831.1 |  | MG548246.1 |  | OU022068.1 |
|  | DQ006996.1 |  | KR867712.1 |  | MW561832.1 |  | MG548247.1 |  | OU022069.1 |
|  | DQ006997.1 |  | KR867713.1 |  | MW561833.1 |  | MG548248.1 |  | MW789889.1 |
|  | DQ006998.1 |  | KR260448.1 |  | MW561834.1 |  | MG548249.1 |  | MW789890.1 |
|  | DQ007000.1 |  | KP213111.1 |  | MW561835.1 |  | MG548250.1 |  | MW789891.1 |
|  | AY684213.1 |  | KP213112.1 |  | MW561836.1 |  | MG548251.1 |  | MW789892.1 |
|  | AY684214.1 |  | KP054955.1 |  | MW561837.1 |  | MG564756.1 |  | MW789893.1 |
|  | AY684215.1 |  | KM201216.1 |  | MW561838.1 |  | MG574357.1 |  | MW789894.1 |
|  |  |  | KM201218.1 |  | MW561839.1 |  | MG015826.1 |  | MW789895.1 |
|  |  |  | KM201219.1 |  | MW561840.1 |  | MG015827.1 |  | MW789896.1 |
|  |  |  | KJ534054.1 |  | MW561841.1 |  | MG015828.1 |  | MW789897.1 |
|  |  |  | KJ534055.1 |  | MW561842.1 |  | MG418832.1 |  | MW789898.1 |
|  |  |  | KJ534056.1 |  | MW561843.1 |  | MG418833.1 |  | MW789899.1 |
|  |  |  | KJ534057.1 |  | MW561844.1 |  | MG418834.1 |  | MW789900.1 |
|  |  |  | KJ534058.1 |  | MW561845.1 |  | MG418835.1 |  | MW789901.1 |
|  |  |  | KJ534059.1 |  | MW561846.1 |  | MG459986.1 |  | MW789902.1 |
|  |  |  | KJ534060.1 |  | MW561847.1 |  | KY393168.1 |  | MW789903.1 |
|  |  |  | KJ534061.1 |  | MW561848.1 |  | KY393169.1 |  | MW789919.1 |
|  |  |  | KJ534062.1 |  | MW561849.1 |  | KY393170.1 |  | MW789920.1 |
|  |  |  | KJ534063.1 |  | MW561850.1 |  | KY393171.1 |  | MW789921.1 |
|  |  |  | KJ561168.1 |  | MW561851.1 |  | KY393172.1 |  | MW789922.1 |
|  |  |  | KF764673.1 |  | MW561852.1 |  | KY393173.1 |  | MW789923.1 |
|  |  |  | KF764674.1 |  | MW561853.1 |  | KY393174.1 |  | MW789924.1 |
|  |  |  | KF764675.1 |  | MW561854.1 |  | KY393175.1 |  | MW789925.1 |
|  |  |  | KF764676.1 |  | MW561855.1 |  | KY701766.1 |  | MW789926.1 |
|  |  |  | KF764677.1 |  | MW561856.1 |  | KY275259.1 |  | MW789927.1 |
|  |  |  | KF764678.1 |  | MW561857.1 |  | KX464811.1 |  | MW789928.1 |
|  |  |  | KF764679.1 |  | MW561858.1 |  | KX464812.1 |  | LC589149.1 |
|  |  |  | KF764680.1 |  | MW561859.1 |  | KX464813.1 |  | MW419259.1 |
|  |  |  | KF764681.1 |  | MW561860.1 |  | KX464814.1 |  | MW419260.1 |
|  |  |  | KF764685.1 |  | MW561861.1 |  | KX464815.1 |  | MW419261.1 |
|  |  |  | KF870480.1 |  | MW561862.1 |  | KX464816.1 |  | MW419262.1 |
|  |  |  | KF870481.1 |  | MW561863.1 |  | KX464817.1 |  | MW419263.1 |
|  |  |  | KF467610.1 |  | MW561864.1 |  | KX464818.1 |  | MW419264.1 |
|  |  |  | KF467611.1 |  | MW561865.1 |  | KX464819.1 |  | MW419265.1 |
|  |  |  | KF467612.1 |  | MW561866.1 |  | KX464820.1 |  | MW419266.1 |
|  |  |  | KF179078.1 |  | MW561867.1 |  | KX464821.1 |  | MW419267.1 |
|  |  |  | KF179079.1 |  | MW561868.1 |  | KX464822.1 |  | MW419268.1 |
|  |  |  | KF179080.1 |  | MW561869.1 |  | KX464823.1 |  | MW419269.1 |
|  |  |  | KF179081.1 |  | MW561870.1 |  | KX464824.1 |  | MW419270.1 |
|  |  |  | KF179082.1 |  | MW561871.1 |  | KX464825.1 |  | MW419271.1 |
|  |  |  | KF179083.1 |  | MW561872.1 |  | KX464826.1 |  | MW419272.1 |
|  |  |  | KF179084.1 |  | MW561873.1 |  | KX464827.1 |  | MW419273.1 |
|  |  |  | KF179085.1 |  | MT370473.1 |  | KX464828.1 |  | MW419274.1 |
|  |  |  | JX962864.1 |  | LC593744.1 |  | KX464829.1 |  | MT897882.1 |
|  |  |  | JX962865.1 |  | LC593747.1 |  | KX464830.1 |  | MT592643.1 |
|  |  |  | JX962866.1 |  | LC593749.1 |  | KX464831.1 |  | MT592644.1 |
|  |  |  | JX962867.1 |  | LC593750.1 |  | KX464832.1 |  | MT592645.1 |
|  |  |  | JX521843.1 |  | LC593751.1 |  | KX464833.1 |  | MT592646.1 |
|  |  |  | JX521841.1 |  | LC593752.1 |  | KX464834.1 |  | MT592647.1 |
|  |  |  | JX521842.1 |  | LC593753.1 |  | KX464835.1 |  | MT592648.1 |
|  |  |  | KC480188.1 |  | LC593754.1 |  | KX464836.1 |  | MT592649.1 |
|  |  |  | KC480187.1 |  | LC593761.1 |  | KX464837.1 |  | MT592650.1 |
|  |  |  | JX515695.1 |  | LC593766.1 |  | KX464838.1 |  | MT592651.1 |
|  |  |  | JX515696.1 |  | LC593771.1 |  | KX464839.1 |  | MT592652.1 |
|  |  |  | JX133240.1 |  | LC585165.1 |  | KX464840.1 |  | MT592709.1 |
|  |  |  | JX133241.1 |  | LC585166.1 |  | KX464841.1 |  | MT592710.1 |
|  |  |  | JX133242.1 |  | LC585167.1 |  | KX464842.1 |  | MT592711.1 |
|  |  |  | JX073101.2 |  | LC585168.1 |  | KX464843.1 |  | MT592712.1 |
|  |  |  | JX073092.1 |  | LC585169.1 |  | KX464844.1 |  | MT592713.1 |
|  |  |  | JX073093.1 |  | LC585170.1 |  | KX464845.1 |  | MT592733.1 |
|  |  |  | JX073102.2 |  | LC585171.1 |  | KX464846.1 |  | MW052272.1 |
|  |  |  | JF934927.1 |  | LC585176.1 |  | KX464847.1 |  | MW052273.1 |
|  |  |  | JF934928.1 |  | LC585177.1 |  | KX259146.1 |  | MW556028.1 |
|  |  |  | JF934929.1 |  | LC585178.1 |  | KX259147.1 |  | MT141110.1 |
|  |  |  | JF934930.1 |  | LC585179.1 |  | KX259148.1 |  | MT120848.1 |
|  |  |  | JF934931.1 |  | LC585180.1 |  | KX259149.1 |  | MT028955.1 |
|  |  |  | JF934932.1 |  | LC585185.1 |  | KX259150.1 |  | MT028956.1 |
|  |  |  | JF934933.1 |  | LC602820.1 |  | KX259151.1 |  | MT028957.1 |
|  |  |  | JF934934.1 |  | MT897881.1 |  | KX259152.1 |  | MT028958.1 |
|  |  |  | JF934935.1 |  | MT592465.1 |  | KX259153.1 |  | MT028959.1 |
|  |  |  | JF934936.1 |  | MT592466.1 |  | KX259154.1 |  | MT028960.1 |
|  |  |  | JF934937.1 |  | MT592467.1 |  | KX259155.1 |  | MT028961.1 |
|  |  |  | JF934938.1 |  | MT592470.1 |  | KX259156.1 |  | MT028962.1 |
|  |  |  | JF934939.1 |  | MT211577.1 |  | KX259157.1 |  | MT028963.1 |
|  |  |  | JF934940.1 |  | MW052274.1 |  | KX259158.1 |  | MT028964.1 |
|  |  |  | JF934941.1 |  | MW052275.1 |  | KX259159.1 |  | MT028965.1 |
|  |  |  | JF934942.1 |  | MW202404.1 |  | KX259160.1 |  | MT028966.1 |
|  |  |  | JF934943.1 |  | MW206378.1 |  | KX259161.1 |  | MT028967.1 |
|  |  |  | JF934944.1 |  | MW147482.1 |  | KX259162.1 |  | MT028968.1 |
|  |  |  | JF934945.1 |  | MT792797.1 |  | KX259163.1 |  | MT028969.1 |
|  |  |  | JF934946.1 |  | MT371067.1 |  | KX259164.1 |  | MT028970.1 |
|  |  |  | JF934947.1 |  | MT371068.1 |  | KX259165.1 |  | MT028971.1 |
|  |  |  | JQ691663.1 |  | MT371069.1 |  | KX259166.1 |  | MT028972.1 |
|  |  |  | JQ691664.1 |  | MT371070.1 |  | KX259167.1 |  | MT028973.1 |
|  |  |  | JQ691665.1 |  | MT371071.1 |  | KX259168.1 |  | MT028974.1 |
|  |  |  | JQ691666.1 |  | MT371072.1 |  | KX259169.1 |  | MT028975.1 |
|  |  |  | JQ691667.1 |  | MT371073.1 |  | KX259170.1 |  | MT028976.1 |
|  |  |  | JQ691668.1 |  | MN537430.1 |  | KX259171.1 |  | MT028977.1 |
|  |  |  | JQ691669.1 |  | MW030649.1 |  | KX259172.1 |  | MT028978.1 |
|  |  |  | JQ691670.1 |  | MN794197.1 |  | KX259173.1 |  | MT028979.1 |
|  |  |  | JQ691671.1 |  | MN794198.1 |  | KX259174.1 |  | MT028980.1 |
|  |  |  | JQ691672.1 |  | MN794199.1 |  | KX259175.1 |  | MT028981.1 |
|  |  |  | JQ691673.1 |  | MN794200.1 |  | KX259176.1 |  | MT028982.1 |
|  |  |  | JQ691674.1 |  | MN794201.1 |  | KX259177.1 |  | MT028983.1 |
|  |  |  | JQ038909.1 |  | MT309728.1 |  | KX259178.1 |  | MT028984.1 |
|  |  |  | JQ038910.1 |  | MT309730.1 |  | KX259179.1 |  | MT028985.1 |
|  |  |  | JQ044459.1 |  | MT309731.1 |  | KX259180.1 |  | MT028986.1 |
|  |  |  | JQ044460.1 |  | MT309732.1 |  | KX259181.1 |  | MT028987.1 |
|  |  |  | JQ044461.1 |  | MT309733.1 |  | KX259182.1 |  | MT028988.1 |
|  |  |  | JQ044462.1 |  | MT309734.1 |  | KX259183.1 |  | MT028989.1 |
|  |  |  | JQ044463.1 |  | MT309735.1 |  | KX138560.1 |  | MT028990.1 |
|  |  |  | JQ044464.1 |  | MT309736.1 |  | KU976444.1 |  | MT028991.1 |
|  |  |  | JQ044465.1 |  | MT309737.1 |  | KU976445.1 |  | MT028992.1 |
|  |  |  | JQ044466.1 |  | MT309739.1 |  | KU976446.1 |  | MT028993.1 |
|  |  |  | JQ044467.1 |  | MT309740.1 |  | KU976447.1 |  | MT028994.1 |
|  |  |  | JQ044468.1 |  | MT309741.1 |  | KU976448.1 |  | MT028995.1 |
|  |  |  | JQ044469.1 |  | MT309742.1 |  | KU976449.1 |  | MT028996.1 |
|  |  |  | JQ044470.1 |  | MN539159.1 |  | KU976450.1 |  | MN536686.1 |
|  |  |  | JQ044471.1 |  | MN539160.1 |  | KU976451.1 |  | MN536687.1 |
|  |  |  | JQ044472.1 |  | MN539161.1 |  | KU976452.1 |  | MN536688.1 |
|  |  |  | JQ044473.1 |  | MN539162.1 |  | KU976453.1 |  | MN536689.1 |
|  |  |  | JQ044474.1 |  | MN539165.1 |  | KU976454.1 |  | MN536690.1 |
|  |  |  | JQ044475.1 |  | MN539166.1 |  | KU065143.1 |  | MN536691.1 |
|  |  |  | JQ044476.1 |  | MN539167.1 |  | KP699088.1 |  | MT309729.1 |
|  |  |  | JQ044477.1 |  | MN539169.1 |  | KP699089.1 |  | MN539190.1 |
|  |  |  | JQ044478.1 |  | MN539175.1 |  | KU198427.1 |  | MN539191.1 |
|  |  |  | JQ044479.1 |  | MT680215.1 |  | KT954161.1 |  | MN839598.1 |
|  |  |  | JQ044480.1 |  | MN867665.1 |  | KT954164.1 |  | MN839599.1 |
|  |  |  | JQ044481.1 |  | MT374083.1 |  | KT954173.1 |  | MT537938.1 |
|  |  |  | JQ044482.1 |  | MN642587.1 |  | KT460190.1 |  | MN708513.1 |
|  |  |  | JQ044483.1 |  | MT454343.1 |  | KT460191.1 |  | MN708514.1 |
|  |  |  | JQ044484.1 |  | MN604260.1 |  | KP721693.1 |  | MN708515.1 |
|  |  |  | JQ044485.1 |  | MN855216.1 |  | KP721694.1 |  | MN708516.1 |
|  |  |  | JQ044486.1 |  | MT454051.1 |  | KP721695.1 |  | MN708517.1 |
|  |  |  | JQ044487.1 |  | MT454052.1 |  | KP721696.1 |  | MN708518.1 |
|  |  |  | JQ044488.1 |  | MT454053.1 |  | KP721697.1 |  | MN708519.1 |
|  |  |  | JQ044489.1 |  | MT454054.1 |  | KP762453.1 |  | MN708520.1 |
|  |  |  | JQ044490.1 |  | MT309378.1 |  | KP762454.1 |  | MN708521.1 |
|  |  |  | JQ044491.1 |  | MT309379.1 |  | KP762455.1 |  | MT424810.1 |
|  |  |  | JQ044492.1 |  | MT309380.1 |  | KP762456.1 |  | MT424811.1 |
|  |  |  | JQ044493.1 |  | MN370930.1 |  | KP762457.1 |  | MT121112.1 |
|  |  |  | JQ044494.1 |  | MN318117.1 |  | KP762458.1 |  | MT121113.1 |
|  |  |  | JQ044495.1 |  | MN318118.1 |  | KP762459.1 |  | MN872683.1 |
|  |  |  | JQ044496.1 |  | MN318119.1 |  | KP762460.1 |  | MN643160.1 |
|  |  |  | JQ044497.1 |  | MN318120.1 |  | KP762461.1 |  | MT454058.1 |
|  |  |  | JQ044498.1 |  | MN515421.1 |  | KP762462.1 |  | MT454059.1 |
|  |  |  | JQ044499.1 |  | MK511445.1 |  | KP762463.1 |  | MN905746.1 |
|  |  |  | JQ044500.1 |  | MK986554.1 |  | KP762464.1 |  | MN905747.1 |
|  |  |  | JQ044501.1 |  | MK986555.1 |  | KP762465.1 |  | MT309399.1 |
|  |  |  | JQ044502.1 |  | MK986556.1 |  | KP762466.1 |  | MT409397.1 |
|  |  |  | JQ044503.1 |  | MK423987.1 |  | KP762467.1 |  | MT015600.1 |
|  |  |  | JQ044504.1 |  | MK423988.1 |  | KP762468.1 |  | MT293532.1 |
|  |  |  | JQ044505.1 |  | MK423989.1 |  | KP762469.1 |  | MT293533.1 |
|  |  |  | JQ044506.1 |  | MK651137.1 |  | KP762470.1 |  | MT293534.1 |
|  |  |  | JQ044507.1 |  | MK651138.1 |  | KP762471.1 |  | MT293535.1 |
|  |  |  | JQ044508.1 |  | MK651139.1 |  | KP762472.1 |  | MN963816.1 |
|  |  |  | JQ044509.1 |  | MK651140.1 |  | KP762473.1 |  | MN850331.1 |
|  |  |  | JQ044510.1 |  | MK651141.1 |  | KP762474.1 |  | MN318108.1 |
|  |  |  | JQ044511.1 |  | MK651142.1 |  | KP762475.1 |  | MN318109.1 |
|  |  |  | JQ044512.1 |  | MK651143.1 |  | KP762476.1 |  | MN318110.1 |
|  |  |  | JQ044513.1 |  | MK651144.1 |  | KP692192.1 |  | MN318111.1 |
|  |  |  | JQ044514.1 |  | MK651145.1 |  | KP692193.1 |  | MN318112.1 |
|  |  |  | JQ044515.1 |  | MK651146.1 |  | KP692194.1 |  | MK412882.1 |
|  |  |  | JQ044516.1 |  | MK651147.1 |  | KP692195.1 |  | MN022786.1 |
|  |  |  | HQ605013.1 |  | MK651148.1 |  | KP692196.1 |  | MN623343.1 |
|  |  |  | HQ605014.1 |  | MK651149.1 |  | KP692197.1 |  | MN623344.1 |
|  |  |  | HQ605015.1 |  | MK651150.1 |  | KP692198.1 |  | MN623345.1 |
|  |  |  | HQ605016.1 |  | MK651151.1 |  | KP692199.1 |  | MN617761.1 |
|  |  |  | HQ605017.1 |  | MK651152.1 |  | KP692200.1 |  | MN119477.1 |
|  |  |  | HQ605018.1 |  | MK651153.1 |  | KP692201.1 |  | MN119478.1 |
|  |  |  | HQ605024.1 |  | MK651154.1 |  | KF955878.1 |  | MK886795.1 |
|  |  |  | JF275874.1 |  | MK651155.1 |  | KF955879.1 |  | MK886796.1 |
|  |  |  | JF275875.1 |  | MK651156.1 |  | KF955880.1 |  | MK952193.1 |
|  |  |  | JF275876.1 |  | MK651157.1 |  | KF778890.1 |  | MK952194.1 |
|  |  |  | JF275877.1 |  | MK651158.1 |  | KF778891.1 |  | MN461162.1 |
|  |  |  | JF275878.1 |  | MK651159.1 |  | KF778892.1 |  | MK675117.1 |
|  |  |  | JF275879.1 |  | MK651160.1 |  | KF778893.1 |  | MK675118.1 |
|  |  |  | JF275884.1 |  | MK651161.1 |  | KF778894.1 |  | MK573997.1 |
|  |  |  | JF275885.1 |  | MK651162.1 |  | KF778895.1 |  | MK573998.1 |
|  |  |  | JF275886.1 |  | MK651163.1 |  | KF778896.1 |  | MK563987.1 |
|  |  |  | JF275887.1 |  | MK651164.1 |  | KF778897.1 |  | MK522098.1 |
|  |  |  | JF275888.1 |  | MK651165.1 |  | KF778898.1 |  | MK522099.1 |
|  |  |  | JF275889.1 |  | MK651166.1 |  | KF778899.1 |  | MK522100.1 |
|  |  |  | HQ159860.1 |  | MK651167.1 |  | KF778900.1 |  | MK522101.1 |
|  |  |  | HQ159861.1 |  | MK651168.1 |  | KF778901.1 |  | MK522102.1 |
|  |  |  | HQ159862.1 |  | MK651169.1 |  | KF778902.1 |  | MK522103.1 |
|  |  |  | HQ159863.1 |  | MK651170.1 |  | KF575087.1 |  | MK522104.1 |
|  |  |  | HQ159864.1 |  | MK651171.1 |  | KF575088.1 |  | MH393617.1 |
|  |  |  | HQ159865.1 |  | MK651172.1 |  | KF575089.1 |  | MH548331.1 |
|  |  |  | HQ159866.1 |  | MK651173.1 |  | KF575090.1 |  | MK524931.1 |
|  |  |  | HQ159867.1 |  | MK651174.1 |  | KF575091.1 |  | MK294085.1 |
|  |  |  | HQ159868.1 |  | MK651175.1 |  | KF575092.1 |  | MK294088.1 |
|  |  |  | HQ159843.1 |  | MK651176.1 |  | KF575093.1 |  | MK294090.1 |
|  |  |  | HQ159844.1 |  | MK651177.1 |  | KJ638974.1 |  | MK252705.1 |
|  |  |  | HQ159845.1 |  | MK651178.1 |  | KF515956.1 |  | LC158109.1 |
|  |  |  | HQ159846.1 |  | MK651179.1 |  | KF515957.1 |  | MH936021.1 |
|  |  |  | HQ159847.1 |  | MK651180.1 |  | KF515958.1 |  | MH521012.1 |
|  |  |  | HQ159848.1 |  | MK651181.1 |  | KF515959.1 |  | MH521013.1 |
|  |  |  | HQ159849.1 |  | MK651182.1 |  | KF515960.1 |  | MH521014.1 |
|  |  |  | HQ159850.1 |  | MK651183.1 |  | KF515966.1 |  | MH800292.1 |
|  |  |  | HQ159851.1 |  | MK651184.1 |  | KF515968.1 |  | MK184989.1 |
|  |  |  | HQ159852.1 |  | MK651185.1 |  | KF481959.1 |  | MK139576.1 |
|  |  |  | HQ159853.1 |  | MK651186.1 |  | KF481960.1 |  | MK088734.1 |
|  |  |  | HQ159854.1 |  | MK651187.1 |  | KF005080.1 |  | MG878231.1 |
|  |  |  | HQ159855.1 |  | MK651188.1 |  | KC960988.1 |  | MG878232.1 |
|  |  |  | HQ159856.1 |  | MK651189.1 |  | KC960989.1 |  | MG878234.1 |
|  |  |  | HQ159857.1 |  | MK651190.1 |  | KC960990.1 |  | MG878237.1 |
|  |  |  | HQ159858.1 |  | MK651191.1 |  | KC960991.1 |  | MG878239.1 |
|  |  |  | HQ159859.1 |  | MK651192.1 |  | KC937065.1 |  | MG878241.1 |
|  |  |  | HQ159869.1 |  | MK651193.1 |  | KC884953.1 |  | MG878243.1 |
|  |  |  | HQ288322.1 |  | MK651194.1 |  | KC440915.1 |  | MG878245.1 |
|  |  |  | HQ288323.1 |  | MK651195.1 |  | JX515676.1 |  | MG878247.1 |
|  |  |  | HQ288324.1 |  | MK651196.1 |  | JX515677.1 |  | MG878252.1 |
|  |  |  | HQ288325.1 |  | MK651197.1 |  | JN607133.1 |  | MG878255.1 |
|  |  |  | HM245321.1 |  | MK651198.1 |  | JN607134.1 |  | MG878257.1 |
|  |  |  | HM245322.1 |  | MK651199.1 |  | JN607135.1 |  | MG878259.1 |
|  |  |  | HM245323.1 |  | MK651200.1 |  | JN607136.1 |  | MG878260.1 |
|  |  |  | HM245324.1 |  | MK651201.1 |  | JX089968.1 |  | MG878262.1 |
|  |  |  | HM245325.1 |  | MK651202.1 |  | JQ659289.1 |  | MG878263.1 |
|  |  |  | GQ903709.1 |  | MK651203.1 |  | JQ659294.1 |  | MG878264.1 |
|  |  |  | GQ903710.1 |  | MK651204.1 |  | JQ659297.1 |  | MG878265.1 |
|  |  |  | GQ903711.1 |  | MK651205.1 |  | JQ659298.1 |  | MG878267.1 |
|  |  |  | GQ903712.1 |  | MK651206.1 |  | JN183860.1 |  | MG878269.1 |
|  |  |  | GQ903713.1 |  | MK651207.1 |  | JN183861.1 |  | MG878272.1 |
|  |  |  | EU863464.1 |  | MK651208.1 |  | JN183862.1 |  | MG878274.1 |
|  |  |  | EU863465.1 |  | MK651209.1 |  | HQ660477.1 |  | MG878275.1 |
|  |  |  | EU863466.1 |  | MK651210.1 |  | HQ288295.1 |  | MG878283.1 |
|  |  |  | EU863467.1 |  | MK651211.1 |  | HQ288296.1 |  | MG878286.1 |
|  |  |  | EU863468.1 |  | MK651212.1 |  | HQ629956.1 |  | MG878287.1 |
|  |  |  | EU863469.1 |  | MK651213.1 |  | HQ629957.1 |  | MG878288.1 |
|  |  |  | EU863470.1 |  | MK651214.1 |  | GU292737.1 |  | MG878292.1 |
|  |  |  | EU863471.1 |  | MK783289.1 |  | GU292748.1 |  | MG878293.1 |
|  |  |  | EU863472.1 |  | MK783290.1 |  | GU292749.1 |  | MG878295.1 |
|  |  |  | EU128062.1 |  | MK783291.1 |  | GU292750.1 |  | MG878298.1 |
|  |  |  | EU128063.1 |  | MK720626.1 |  | GU292751.1 |  | MG878301.1 |
|  |  |  | EU128064.1 |  | MK704424.1 |  | GU292771.1 |  | MG745804.1 |
|  |  |  | EU128065.1 |  | MN508257.1 |  | GU292779.1 |  | MH697825.1 |
|  |  |  | EU128066.1 |  | MN165110.1 |  | GU292792.1 |  | MG649077.1 |
|  |  |  | EU128067.1 |  | MK522082.1 |  | GU251769.1 |  | MH221122.1 |
|  |  |  | EU128068.1 |  | MK522083.1 |  | GU251771.1 |  | MH221123.1 |
|  |  |  | DQ173094.1 |  | MK522084.1 |  | GU251772.1 |  | MG970279.1 |
|  |  |  | DQ173095.1 |  | MK522085.1 |  | GU251773.1 |  | MG970280.1 |
|  |  |  | AF246806.1 |  | MK482381.1 |  | GU251775.1 |  | MG970281.1 |
|  |  |  | AF246807.1 |  | MK482382.1 |  | GU294722.1 |  | MG970282.1 |
|  |  |  | AF246808.1 |  | MN244680.1 |  | GU121821.1 |  | MG970283.1 |
|  |  |  | AF246811.1 |  | MH891168.1 |  | GU121822.1 |  | MG970284.1 |
|  |  |  | AF246812.1 |  | MH726145.1 |  | GU121823.1 |  | MG958628.1 |
|  |  |  | AF246813.1 |  | MH726146.1 |  | GU121824.1 |  | MG958629.1 |
|  |  |  | AF192390.1 |  | MH726147.1 |  | GU121825.1 |  | MG958630.1 |
|  |  |  |  |  | MH726148.1 |  | GU121826.1 |  | MG970289.1 |
|  |  |  |  |  | MH726149.1 |  | GU121827.1 |  | MG970290.1 |
|  |  |  |  |  | MH726150.1 |  | GU121828.1 |  | MG970291.1 |
|  |  |  |  |  | MH726151.1 |  | GU121829.1 |  | MH118939.1 |
|  |  |  |  |  | MH726152.1 |  | GU121830.1 |  | MH423579.1 |
|  |  |  |  |  | MH726153.1 |  | GU121831.1 |  | MF774643.1 |
|  |  |  |  |  | MH726154.1 |  | GU121832.1 |  | MF774650.1 |
|  |  |  |  |  | MH726155.1 |  | GU121833.1 |  | MF631023.1 |
|  |  |  |  |  | MH724212.1 |  | GU121834.1 |  | MF631024.1 |
|  |  |  |  |  | MH521009.1 |  | GU121835.1 |  | MG879024.1 |
|  |  |  |  |  | MH521010.1 |  | EU012429.1 |  | MG879025.1 |
|  |  |  |  |  | MH521011.1 |  | EU012430.1 |  | MF314156.1 |
|  |  |  |  |  | MH791314.1 |  | EU012431.1 |  | MF314157.1 |
|  |  |  |  |  | MG878233.1 |  | EU012432.1 |  | MF314158.1 |
|  |  |  |  |  | MG878236.1 |  | EU012433.1 |  | MF314159.1 |
|  |  |  |  |  | MG878238.1 |  | EU012434.1 |  | MF314160.1 |
|  |  |  |  |  | MG878240.1 |  | EU220473.1 |  | MF314161.1 |
|  |  |  |  |  | MG878242.1 |  | EU220475.1 |  | MF314162.1 |
|  |  |  |  |  | MG878244.1 |  | EU220478.1 |  | MF314163.1 |
|  |  |  |  |  | MG878246.1 |  | EU220479.1 |  | MF314164.1 |
|  |  |  |  |  | MG878248.1 |  | DQ458856.1 |  | MF314165.1 |
|  |  |  |  |  | MG878249.1 |  | DQ458857.1 |  | MG775275.1 |
|  |  |  |  |  | MG878250.1 |  | DQ356361.1 |  | KY000099.1 |
|  |  |  |  |  | MG878251.1 |  | DQ356362.1 |  | KY000122.1 |
|  |  |  |  |  | MG878253.1 |  | DQ356363.1 |  | KY000130.1 |
|  |  |  |  |  | MG878256.1 |  | DQ356364.1 |  | KY000131.1 |
|  |  |  |  |  | MG878258.1 |  | DQ008337.1 |  | KY000154.1 |
|  |  |  |  |  | MG878261.1 |  | DQ008338.1 |  | KY000173.1 |
|  |  |  |  |  | MG878266.1 |  | DQ008339.1 |  | KY000182.1 |
|  |  |  |  |  | MG878268.1 |  | DQ008340.1 |  | KY000201.1 |
|  |  |  |  |  | MG878270.1 |  | DQ008341.1 |  | KY000108.1 |
|  |  |  |  |  | MG878271.1 |  | DQ008342.1 |  | KY000117.1 |
|  |  |  |  |  | MG878273.1 |  | DQ008343.1 |  | KY000128.1 |
|  |  |  |  |  | MG878276.1 |  | DQ008344.1 |  | KY000129.1 |
|  |  |  |  |  | MG878280.1 |  | DQ008345.1 |  | KY000132.1 |
|  |  |  |  |  | MG878284.1 |  | AY972119.1 |  | KY000133.1 |
|  |  |  |  |  | MG878289.1 |  | AY972120.1 |  | KY000135.1 |
|  |  |  |  |  | MG878291.1 |  | AY236931.1 |  | KY000170.1 |
|  |  |  |  |  | MG878294.1 |  | AY236932.1 |  | KY000192.1 |
|  |  |  |  |  | MG878297.1 |  |  |  | KY000213.1 |
|  |  |  |  |  | MG878299.1 |  |  |  | KY000214.1 |
|  |  |  |  |  | MG761773.1 |  |  |  | KX278264.1 |
|  |  |  |  |  | MF597794.1 |  |  |  | KX278265.1 |
|  |  |  |  |  | MG970287.1 |  |  |  | KX278266.1 |
|  |  |  |  |  | MG970288.1 |  |  |  | KX278267.1 |
|  |  |  |  |  | MG958632.1 |  |  |  | KX278268.1 |
|  |  |  |  |  | MH118935.1 |  |  |  | KX278269.1 |
|  |  |  |  |  | MH483927.1 |  |  |  | KX587517.1 |
|  |  |  |  |  | MG564757.1 |  |  |  | KY009943.1 |
|  |  |  |  |  | MG564758.1 |  |  |  | KU997570.1 |
|  |  |  |  |  | MG564759.1 |  |  |  | KU997591.1 |
|  |  |  |  |  | MG564760.1 |  |  |  | KU997617.1 |
|  |  |  |  |  | MG564761.1 |  |  |  | KU997618.1 |
|  |  |  |  |  | MG564762.1 |  |  |  | KU997627.1 |
|  |  |  |  |  | KY978574.1 |  |  |  | KU997567.1 |
|  |  |  |  |  | KY393147.1 |  |  |  | KU997569.1 |
|  |  |  |  |  | KY393148.1 |  |  |  | KU997577.1 |
|  |  |  |  |  | KY393149.1 |  |  |  | KU997578.1 |
|  |  |  |  |  | KY393150.1 |  |  |  | KU997580.1 |
|  |  |  |  |  | KY393151.1 |  |  |  | KU997581.1 |
|  |  |  |  |  | KY393152.1 |  |  |  | KU997582.1 |
|  |  |  |  |  | KY393153.1 |  |  |  | KU997583.1 |
|  |  |  |  |  | KY393154.1 |  |  |  | KU997584.1 |
|  |  |  |  |  | KY393155.1 |  |  |  | KU997585.1 |
|  |  |  |  |  | KY393156.1 |  |  |  | KU997586.1 |
|  |  |  |  |  | KY393157.1 |  |  |  | KU997587.1 |
|  |  |  |  |  | KY393158.1 |  |  |  | KU997589.1 |
|  |  |  |  |  | KY393159.1 |  |  |  | KU997590.1 |
|  |  |  |  |  | KY393160.1 |  |  |  | KU997593.1 |
|  |  |  |  |  | KY393161.1 |  |  |  | KU997594.1 |
|  |  |  |  |  | KY393162.1 |  |  |  | KU997595.1 |
|  |  |  |  |  | KY393163.1 |  |  |  | KU997596.1 |
|  |  |  |  |  | KY393164.1 |  |  |  | KU997597.1 |
|  |  |  |  |  | KY393165.1 |  |  |  | KU997600.1 |
|  |  |  |  |  | KY393166.1 |  |  |  | KU997603.1 |
|  |  |  |  |  | KY393167.1 |  |  |  | KU997606.1 |
|  |  |  |  |  | KU997568.1 |  |  |  | KU997607.1 |
|  |  |  |  |  | KU997579.1 |  |  |  | KU997609.1 |
|  |  |  |  |  | KU997616.1 |  |  |  | KU997610.1 |
|  |  |  |  |  | KX306956.1 |  |  |  | KU997611.1 |
|  |  |  |  |  | KX306957.1 |  |  |  | KU997612.1 |
|  |  |  |  |  | KX306958.1 |  |  |  | KU997613.1 |
|  |  |  |  |  | KX306959.1 |  |  |  | KU997614.1 |
|  |  |  |  |  | KX306960.1 |  |  |  | KU997615.1 |
|  |  |  |  |  | KX648461.1 |  |  |  | KU997619.1 |
|  |  |  |  |  | KX648462.1 |  |  |  | KU997620.1 |
|  |  |  |  |  | KX648463.1 |  |  |  | KU997621.1 |
|  |  |  |  |  | KX648464.1 |  |  |  | KU997622.1 |
|  |  |  |  |  | KX648465.1 |  |  |  | KU997623.1 |
|  |  |  |  |  | KX648466.1 |  |  |  | KU997624.1 |
|  |  |  |  |  | KX648467.1 |  |  |  | KU997625.1 |
|  |  |  |  |  | KX648468.1 |  |  |  | KU997626.1 |
|  |  |  |  |  | KX648469.1 |  |  |  | KU997629.1 |
|  |  |  |  |  | KX648470.1 |  |  |  | KU997630.1 |
|  |  |  |  |  | KX648471.1 |  |  |  | KU997631.1 |
|  |  |  |  |  | KX091155.1 |  |  |  | KU997632.1 |
|  |  |  |  |  | KX464781.1 |  |  |  | KX871766.1 |
|  |  |  |  |  | KU928260.1 |  |  |  | KX871767.1 |
|  |  |  |  |  | KX357149.1 |  |  |  | KX871768.1 |
|  |  |  |  |  | KX357150.1 |  |  |  | KX871769.1 |
|  |  |  |  |  | KX357151.1 |  |  |  | KX871770.1 |
|  |  |  |  |  | KX357152.1 |  |  |  | KX871771.1 |
|  |  |  |  |  | KX357153.1 |  |  |  | KX871772.1 |
|  |  |  |  |  | KU554659.1 |  |  |  | KU530157.1 |
|  |  |  |  |  | KU554660.1 |  |  |  | KX648450.1 |
|  |  |  |  |  | KU554661.1 |  |  |  | KX648451.1 |
|  |  |  |  |  | KU554662.1 |  |  |  | KX648452.1 |
|  |  |  |  |  | KU565871.1 |  |  |  | KX648453.1 |
|  |  |  |  |  | KX281147.1 |  |  |  | KX648454.1 |
|  |  |  |  |  | KR261727.1 |  |  |  | KX648455.1 |
|  |  |  |  |  | KR261728.1 |  |  |  | KX648456.1 |
|  |  |  |  |  | KR261729.1 |  |  |  | KX648457.1 |
|  |  |  |  |  | KR261730.1 |  |  |  | KX648458.1 |
|  |  |  |  |  | KR261731.1 |  |  |  | KX648459.1 |
|  |  |  |  |  | KR261732.1 |  |  |  | KX648460.1 |
|  |  |  |  |  | KR261733.1 |  |  |  | LC120827.1 |
|  |  |  |  |  | KR261734.1 |  |  |  | KX464961.1 |
|  |  |  |  |  | KU306116.1 |  |  |  | KX464962.1 |
|  |  |  |  |  | LC120690.1 |  |  |  | KX464963.1 |
|  |  |  |  |  | KP183117.1 |  |  |  | KX464975.1 |
|  |  |  |  |  | KP183118.1 |  |  |  | KX464976.1 |
|  |  |  |  |  | KP183119.1 |  |  |  | KX464977.1 |
|  |  |  |  |  | KP183120.1 |  |  |  | KX464978.1 |
|  |  |  |  |  | KP183121.1 |  |  |  | KX464979.1 |
|  |  |  |  |  | KP183122.1 |  |  |  | KX464980.1 |
|  |  |  |  |  | KP183123.1 |  |  |  | KX464981.1 |
|  |  |  |  |  | KP183124.1 |  |  |  | KX464982.1 |
|  |  |  |  |  | KP183125.1 |  |  |  | KX464983.1 |
|  |  |  |  |  | KP183126.1 |  |  |  | KX464984.1 |
|  |  |  |  |  | KP183127.1 |  |  |  | KX464985.1 |
|  |  |  |  |  | KP183128.1 |  |  |  | KX464986.1 |
|  |  |  |  |  | KP183129.1 |  |  |  | KX464987.1 |
|  |  |  |  |  | KP183130.1 |  |  |  | KX464988.1 |
|  |  |  |  |  | KP183131.1 |  |  |  | KX464989.1 |
|  |  |  |  |  | KP183132.1 |  |  |  | KX464990.1 |
|  |  |  |  |  | KP183133.1 |  |  |  | KX464991.1 |
|  |  |  |  |  | KP183134.1 |  |  |  | KX464992.1 |
|  |  |  |  |  | KP183135.1 |  |  |  | KX464993.1 |
|  |  |  |  |  | KP183136.1 |  |  |  | KX464994.1 |
|  |  |  |  |  | KP183137.1 |  |  |  | KX464995.1 |
|  |  |  |  |  | KP183138.1 |  |  |  | KX464996.1 |
|  |  |  |  |  | KT581218.1 |  |  |  | KX464997.1 |
|  |  |  |  |  | KT581219.1 |  |  |  | KX465009.1 |
|  |  |  |  |  | KT581220.1 |  |  |  | KX465012.1 |
|  |  |  |  |  | KT581221.1 |  |  |  | KX154807.1 |
|  |  |  |  |  | KT581222.1 |  |  |  | KU554656.1 |
|  |  |  |  |  | KT581223.1 |  |  |  | KU554657.1 |
|  |  |  |  |  | KT581224.1 |  |  |  | KU554658.1 |
|  |  |  |  |  | KT581225.1 |  |  |  | KU984493.1 |
|  |  |  |  |  | KT581226.1 |  |  |  | KU587871.1 |
|  |  |  |  |  | KT581227.1 |  |  |  | KP860771.1 |
|  |  |  |  |  | KT581228.1 |  |  |  | KP860772.1 |
|  |  |  |  |  | KT581229.1 |  |  |  | KP860773.1 |
|  |  |  |  |  | KT581230.1 |  |  |  | KP860774.1 |
|  |  |  |  |  | KT581231.1 |  |  |  | KP860794.1 |
|  |  |  |  |  | KT581232.1 |  |  |  | KP860796.1 |
|  |  |  |  |  | KT581233.1 |  |  |  | KP860805.1 |
|  |  |  |  |  | KT581234.1 |  |  |  | KP860811.1 |
|  |  |  |  |  | KT581235.1 |  |  |  | KP860815.1 |
|  |  |  |  |  | KT935322.1 |  |  |  | KP860817.1 |
|  |  |  |  |  | KT253571.1 |  |  |  | KP860821.1 |
|  |  |  |  |  | KT189503.1 |  |  |  | KP183103.1 |
|  |  |  |  |  | KT189504.1 |  |  |  | KP183107.1 |
|  |  |  |  |  | KR232673.1 |  |  |  | KP183109.1 |
|  |  |  |  |  | KR232675.1 |  |  |  | KP183112.1 |
|  |  |  |  |  | KR232677.1 |  |  |  | KP183100.1 |
|  |  |  |  |  | KR232678.1 |  |  |  | KP183101.1 |
|  |  |  |  |  | KR232679.1 |  |  |  | KP183102.1 |
|  |  |  |  |  | KT026968.1 |  |  |  | KP183104.1 |
|  |  |  |  |  | KR260832.1 |  |  |  | KP183105.1 |
|  |  |  |  |  | KR260833.1 |  |  |  | KP183106.1 |
|  |  |  |  |  | KP721688.1 |  |  |  | KP183108.1 |
|  |  |  |  |  | KP721689.1 |  |  |  | KP183110.1 |
|  |  |  |  |  | KP721690.1 |  |  |  | KP183111.1 |
|  |  |  |  |  | KP721691.1 |  |  |  | KP183113.1 |
|  |  |  |  |  | KP721692.1 |  |  |  | KP183114.1 |
|  |  |  |  |  | KF955864.1 |  |  |  | KP183115.1 |
|  |  |  |  |  | KF955865.1 |  |  |  | KP183116.1 |
|  |  |  |  |  | KF955866.1 |  |  |  | KP699090.1 |
|  |  |  |  |  | KF955867.1 |  |  |  | KT935321.1 |
|  |  |  |  |  | KF955868.1 |  |  |  | KT935320.1 |
|  |  |  |  |  | KF955869.1 |  |  |  | KM103187.1 |
|  |  |  |  |  | KF955870.1 |  |  |  | KM103188.1 |
|  |  |  |  |  | KF955871.1 |  |  |  | KM103199.1 |
|  |  |  |  |  | KF955872.1 |  |  |  | KM103200.1 |
|  |  |  |  |  | KF955873.1 |  |  |  | KM103201.1 |
|  |  |  |  |  | KF955874.1 |  |  |  | KT189502.1 |
|  |  |  |  |  | KF955875.1 |  |  |  | KR232676.1 |
|  |  |  |  |  | KF955876.1 |  |  |  | KR260807.1 |
|  |  |  |  |  | KF955877.1 |  |  |  | KP721702.1 |
|  |  |  |  |  | KJ863647.1 |  |  |  | KP721703.1 |
|  |  |  |  |  | KJ863648.1 |  |  |  | KP721704.1 |
|  |  |  |  |  | KJ863649.1 |  |  |  | KP721705.1 |
|  |  |  |  |  | KJ863650.1 |  |  |  | KP721706.1 |
|  |  |  |  |  | KJ863651.1 |  |  |  | KP762482.1 |
|  |  |  |  |  | KJ863652.1 |  |  |  | KP762483.1 |
|  |  |  |  |  | KJ863653.1 |  |  |  | KP762484.1 |
|  |  |  |  |  | KJ801793.1 |  |  |  | KP762485.1 |
|  |  |  |  |  | KF778878.1 |  |  |  | KF955950.1 |
|  |  |  |  |  | KF778879.1 |  |  |  | KF955951.1 |
|  |  |  |  |  | KF778880.1 |  |  |  | KP190148.1 |
|  |  |  |  |  | KF778881.1 |  |  |  | KF923263.1 |
|  |  |  |  |  | KF778882.1 |  |  |  | KF923264.1 |
|  |  |  |  |  | KF778883.1 |  |  |  | KF923265.1 |
|  |  |  |  |  | KF575102.1 |  |  |  | KJ946461.1 |
|  |  |  |  |  | KF575103.1 |  |  |  | KJ946462.1 |
|  |  |  |  |  | KF575104.1 |  |  |  | KJ841779.1 |
|  |  |  |  |  | KJ530707.1 |  |  |  | KF778949.1 |
|  |  |  |  |  | KF005077.1 |  |  |  | KF778950.1 |
|  |  |  |  |  | KF005078.1 |  |  |  | KF778951.1 |
|  |  |  |  |  | KC960993.1 |  |  |  | KF778952.1 |
|  |  |  |  |  | KC961003.1 |  |  |  | KF778953.1 |
|  |  |  |  |  | KC961026.1 |  |  |  | KF778954.1 |
|  |  |  |  |  | KC960994.1 |  |  |  | KF778955.1 |
|  |  |  |  |  | KC960995.1 |  |  |  | KF778956.1 |
|  |  |  |  |  | KC960996.1 |  |  |  | KF778957.1 |
|  |  |  |  |  | KC960997.1 |  |  |  | KF778958.1 |
|  |  |  |  |  | KC960998.1 |  |  |  | KF778959.1 |
|  |  |  |  |  | KC960999.1 |  |  |  | KF778960.1 |
|  |  |  |  |  | KC961000.1 |  |  |  | KF778961.1 |
|  |  |  |  |  | KC961001.1 |  |  |  | KF778962.1 |
|  |  |  |  |  | KC961002.1 |  |  |  | KF575113.1 |
|  |  |  |  |  | KC961004.1 |  |  |  | KF575114.1 |
|  |  |  |  |  | KC961005.1 |  |  |  | KF575115.1 |
|  |  |  |  |  | KC961006.1 |  |  |  | KF575116.1 |
|  |  |  |  |  | KC961007.1 |  |  |  | KF575117.1 |
|  |  |  |  |  | KC961008.1 |  |  |  | KF575118.1 |
|  |  |  |  |  | KC961009.1 |  |  |  | KF886719.1 |
|  |  |  |  |  | KC961010.1 |  |  |  | KF886720.1 |
|  |  |  |  |  | KC961011.1 |  |  |  | KF886721.1 |
|  |  |  |  |  | KC961012.1 |  |  |  | KF886722.1 |
|  |  |  |  |  | KC961013.1 |  |  |  | KC794027.1 |
|  |  |  |  |  | KC961014.1 |  |  |  | KC794028.1 |
|  |  |  |  |  | KC961015.1 |  |  |  | KC794029.1 |
|  |  |  |  |  | KC961016.1 |  |  |  | KF729324.1 |
|  |  |  |  |  | KC961017.1 |  |  |  | KF729325.1 |
|  |  |  |  |  | KC961018.1 |  |  |  | KF729326.1 |
|  |  |  |  |  | KC961019.1 |  |  |  | KF729327.1 |
|  |  |  |  |  | KC961020.1 |  |  |  | KF729328.1 |
|  |  |  |  |  | KC961021.1 |  |  |  | KF729329.1 |
|  |  |  |  |  | KC961022.1 |  |  |  | KF729330.1 |
|  |  |  |  |  | KC961023.1 |  |  |  | KF729331.1 |
|  |  |  |  |  | KC961024.1 |  |  |  | KF729332.1 |
|  |  |  |  |  | KC961025.1 |  |  |  | KF729333.1 |
|  |  |  |  |  | KC961027.1 |  |  |  | KF729334.1 |
|  |  |  |  |  | KC961028.1 |  |  |  | KF729335.1 |
|  |  |  |  |  | KC937063.1 |  |  |  | KF729336.1 |
|  |  |  |  |  | KC864747.2 |  |  |  | KF729337.1 |
|  |  |  |  |  | KC769890.1 |  |  |  | KF729338.1 |
|  |  |  |  |  | KC769891.1 |  |  |  | KF729339.1 |
|  |  |  |  |  | KC769892.1 |  |  |  | KF729340.1 |
|  |  |  |  |  | KC769898.1 |  |  |  | KF729341.1 |
|  |  |  |  |  | KC769899.1 |  |  |  | KF729342.1 |
|  |  |  |  |  | KC769900.1 |  |  |  | KF729343.1 |
|  |  |  |  |  | KC440913.1 |  |  |  | KF729344.1 |
|  |  |  |  |  | JX462258.1 |  |  |  | KF729345.1 |
|  |  |  |  |  | JX462259.1 |  |  |  | KF729346.1 |
|  |  |  |  |  | JX462260.1 |  |  |  | KF729347.1 |
|  |  |  |  |  | JX462261.1 |  |  |  | KF729348.1 |
|  |  |  |  |  | JX462264.1 |  |  |  | KF729349.1 |
|  |  |  |  |  | JX462266.1 |  |  |  | KF729350.1 |
|  |  |  |  |  | JX462267.1 |  |  |  | KF729351.1 |
|  |  |  |  |  | JX462268.1 |  |  |  | KF729352.1 |
|  |  |  |  |  | JX462269.1 |  |  |  | KF729353.1 |
|  |  |  |  |  | JX462270.1 |  |  |  | KF729354.1 |
|  |  |  |  |  | JX462271.1 |  |  |  | KF729355.1 |
|  |  |  |  |  | JX462273.1 |  |  |  | KF729356.1 |
|  |  |  |  |  | JX462274.1 |  |  |  | KF729357.1 |
|  |  |  |  |  | JX462275.1 |  |  |  | KF729358.1 |
|  |  |  |  |  | JX462277.1 |  |  |  | KF729359.1 |
|  |  |  |  |  | JX462279.1 |  |  |  | KF729360.1 |
|  |  |  |  |  | JX462280.1 |  |  |  | KF729361.1 |
|  |  |  |  |  | JX462281.1 |  |  |  | KF729362.1 |
|  |  |  |  |  | JX515667.1 |  |  |  | KF729363.1 |
|  |  |  |  |  | JX515668.1 |  |  |  | KF729484.1 |
|  |  |  |  |  | JX556419.1 |  |  |  | KC507806.1 |
|  |  |  |  |  | JX392807.1 |  |  |  | KC507808.1 |
|  |  |  |  |  | JN607130.1 |  |  |  | KF454706.1 |
|  |  |  |  |  | JN607131.1 |  |  |  | KF515963.1 |
|  |  |  |  |  | JN607132.1 |  |  |  | KF515953.1 |
|  |  |  |  |  | JN607154.1 |  |  |  | KF515954.1 |
|  |  |  |  |  | JX089972.1 |  |  |  | KF515955.1 |
|  |  |  |  |  | JQ411422.1 |  |  |  | KF005079.1 |
|  |  |  |  |  | JQ900375.1 |  |  |  | KC960985.1 |
|  |  |  |  |  | JQ403583.1 |  |  |  | KC960986.1 |
|  |  |  |  |  | HM176484.1 |  |  |  | KC960987.1 |
|  |  |  |  |  | HM176485.1 |  |  |  | KC884952.1 |
|  |  |  |  |  | JQ080549.1 |  |  |  | KC769874.1 |
|  |  |  |  |  | JF440812.1 |  |  |  | KC769875.1 |
|  |  |  |  |  | JF440813.1 |  |  |  | KC769876.1 |
|  |  |  |  |  | JF440814.1 |  |  |  | KC769901.1 |
|  |  |  |  |  | JF440815.1 |  |  |  | JX462257.1 |
|  |  |  |  |  | JF440816.1 |  |  |  | JX462263.1 |
|  |  |  |  |  | JF440817.1 |  |  |  | JX462272.1 |
|  |  |  |  |  | JF440818.1 |  |  |  | KC631653.1 |
|  |  |  |  |  | JF440819.1 |  |  |  | KC631654.1 |
|  |  |  |  |  | JF440820.1 |  |  |  | KC621052.1 |
|  |  |  |  |  | JF440821.1 |  |  |  | KC621053.1 |
|  |  |  |  |  | JF440822.1 |  |  |  | KC621054.1 |
|  |  |  |  |  | JF440823.1 |  |  |  | KC621055.1 |
|  |  |  |  |  | JF440824.1 |  |  |  | KC621056.1 |
|  |  |  |  |  | JF440825.1 |  |  |  | KC621057.1 |
|  |  |  |  |  | JF440826.1 |  |  |  | KC621058.1 |
|  |  |  |  |  | JF440827.1 |  |  |  | KC621059.1 |
|  |  |  |  |  | JF440828.1 |  |  |  | KC621060.1 |
|  |  |  |  |  | JF440829.1 |  |  |  | KC621061.1 |
|  |  |  |  |  | JF440830.1 |  |  |  | KC621062.1 |
|  |  |  |  |  | JF440831.1 |  |  |  | KC621063.1 |
|  |  |  |  |  | JF440832.1 |  |  |  | JX646843.1 |
|  |  |  |  |  | JF440833.1 |  |  |  | JX392805.1 |
|  |  |  |  |  | JF440834.1 |  |  |  | JX392806.2 |
|  |  |  |  |  | JF440835.1 |  |  |  | JX398944.1 |
|  |  |  |  |  | JF440837.1 |  |  |  | JQ918158.1 |
|  |  |  |  |  | JF440838.1 |  |  |  | JQ411452.1 |
|  |  |  |  |  | JF440839.1 |  |  |  | JQ659292.1 |
|  |  |  |  |  | JF440840.1 |  |  |  | JQ659293.1 |
|  |  |  |  |  | JF440841.1 |  |  |  | JQ659300.1 |
|  |  |  |  |  | JF440842.1 |  |  |  | JQ974954.1 |
|  |  |  |  |  | JF440843.1 |  |  |  | HQ859953.1 |
|  |  |  |  |  | JF440844.1 |  |  |  | HQ859954.1 |
|  |  |  |  |  | JF440845.1 |  |  |  | HQ840417.1 |
|  |  |  |  |  | JF440846.1 |  |  |  | JN811824.1 |
|  |  |  |  |  | JF440847.1 |  |  |  | JQ080552.1 |
|  |  |  |  |  | JF440848.1 |  |  |  | JN595815.1 |
|  |  |  |  |  | JF440849.1 |  |  |  | JN595819.1 |
|  |  |  |  |  | JF440850.1 |  |  |  | JN595823.1 |
|  |  |  |  |  | JF440851.1 |  |  |  | HQ529734.1 |
|  |  |  |  |  | JF440852.1 |  |  |  | HQ529735.1 |
|  |  |  |  |  | JF501228.1 |  |  |  | HQ529736.1 |
|  |  |  |  |  | JF682849.1 |  |  |  | HQ529737.1 |
|  |  |  |  |  | HQ529720.1 |  |  |  | HQ529739.1 |
|  |  |  |  |  | HQ529721.1 |  |  |  | HQ529740.1 |
|  |  |  |  |  | HQ529723.1 |  |  |  | JN191296.1 |
|  |  |  |  |  | HQ529722.2 |  |  |  | JN191297.1 |
|  |  |  |  |  | JN183856.1 |  |  |  | JN191298.1 |
|  |  |  |  |  | JF441078.1 |  |  |  | HM545151.1 |
|  |  |  |  |  | JF441079.1 |  |  |  | HM545152.1 |
|  |  |  |  |  | JF441080.1 |  |  |  | HM545153.1 |
|  |  |  |  |  | JF441081.1 |  |  |  | HM545154.1 |
|  |  |  |  |  | JF441082.1 |  |  |  | JF271780.1 |
|  |  |  |  |  | JF441083.1 |  |  |  | HQ392764.1 |
|  |  |  |  |  | HQ660465.1 |  |  |  | HQ392766.1 |
|  |  |  |  |  | HQ660466.1 |  |  |  | HM480386.1 |
|  |  |  |  |  | HQ660467.1 |  |  |  | HM480387.1 |
|  |  |  |  |  | HQ660468.1 |  |  |  | GU292752.1 |
|  |  |  |  |  | HQ660469.1 |  |  |  | GU292753.1 |
|  |  |  |  |  | HQ660470.1 |  |  |  | GU292770.1 |
|  |  |  |  |  | HQ660471.1 |  |  |  | GU292787.1 |
|  |  |  |  |  | HQ660472.1 |  |  |  | GU292788.1 |
|  |  |  |  |  | HQ660473.1 |  |  |  | GU251783.1 |
|  |  |  |  |  | HQ660475.1 |  |  |  | GU251784.1 |
|  |  |  |  |  | HQ660476.1 |  |  |  | GU251785.1 |
|  |  |  |  |  | HQ288289.1 |  |  |  | GU251789.1 |
|  |  |  |  |  | HQ288290.1 |  |  |  | GU251790.1 |
|  |  |  |  |  | HQ392738.1 |  |  |  | GU251791.1 |
|  |  |  |  |  | HQ392743.1 |  |  |  | GU251792.1 |
|  |  |  |  |  | HQ392745.1 |  |  |  | GU251793.1 |
|  |  |  |  |  | HQ392748.1 |  |  |  | GU251794.1 |
|  |  |  |  |  | HQ392756.1 |  |  |  | GU251795.1 |
|  |  |  |  |  | HQ392758.1 |  |  |  | GU251796.1 |
|  |  |  |  |  | GU292738.1 |  |  |  | GU251797.1 |
|  |  |  |  |  | GU292739.1 |  |  |  | GU251798.1 |
|  |  |  |  |  | GU292740.1 |  |  |  | GU251799.1 |
|  |  |  |  |  | GU292741.1 |  |  |  | GU251800.1 |
|  |  |  |  |  | GU292742.1 |  |  |  | GU251801.1 |
|  |  |  |  |  | GU292743.1 |  |  |  | GU251802.1 |
|  |  |  |  |  | GU292744.1 |  |  |  | GU251803.1 |
|  |  |  |  |  | GU292745.1 |  |  |  | GU251804.1 |
|  |  |  |  |  | GU292746.1 |  |  |  | GU251805.1 |
|  |  |  |  |  | GU292747.1 |  |  |  | GU251806.1 |
|  |  |  |  |  | GU292760.1 |  |  |  | GU997687.1 |
|  |  |  |  |  | GU292761.1 |  |  |  | GU294723.1 |
|  |  |  |  |  | GU292762.1 |  |  |  | GU121836.1 |
|  |  |  |  |  | GU292763.1 |  |  |  | GU121837.1 |
|  |  |  |  |  | GU292764.1 |  |  |  | GU121838.1 |
|  |  |  |  |  | GU292765.1 |  |  |  | GU121839.1 |
|  |  |  |  |  | GU292766.1 |  |  |  | GU121840.1 |
|  |  |  |  |  | GU292767.1 |  |  |  | GU121841.1 |
|  |  |  |  |  | GU292768.1 |  |  |  | GU121842.1 |
|  |  |  |  |  | GU292769.1 |  |  |  | GU121843.1 |
|  |  |  |  |  | GU292772.1 |  |  |  | GU121844.1 |
|  |  |  |  |  | GU292773.1 |  |  |  | GU121845.1 |
|  |  |  |  |  | GU292774.1 |  |  |  | GU121846.1 |
|  |  |  |  |  | GU292777.1 |  |  |  | GU121847.1 |
|  |  |  |  |  | GU292778.1 |  |  |  | FJ900637.1 |
|  |  |  |  |  | GU251751.1 |  |  |  | FJ900638.1 |
|  |  |  |  |  | GU251752.1 |  |  |  | FJ900639.1 |
|  |  |  |  |  | GU251753.1 |  |  |  | FJ900640.1 |
|  |  |  |  |  | GU251754.1 |  |  |  | GQ857666.1 |
|  |  |  |  |  | GU251755.1 |  |  |  | GQ857667.1 |
|  |  |  |  |  | GU251756.1 |  |  |  | EU821842.1 |
|  |  |  |  |  | GU251757.1 |  |  |  | EU821847.1 |
|  |  |  |  |  | GU251758.1 |  |  |  | EU821848.1 |
|  |  |  |  |  | GU251759.1 |  |  |  | EU821849.1 |
|  |  |  |  |  | GU251760.1 |  |  |  | EU821850.1 |
|  |  |  |  |  | GU251761.1 |  |  |  | EU821851.1 |
|  |  |  |  |  | GU251762.1 |  |  |  | EU821852.1 |
|  |  |  |  |  | GU251763.1 |  |  |  | EU821854.1 |
|  |  |  |  |  | GU251764.1 |  |  |  | EU821856.1 |
|  |  |  |  |  | GU997689.1 |  |  |  | EU821857.1 |
|  |  |  |  |  | GU294728.1 |  |  |  | EU821858.1 |
|  |  |  |  |  | GQ857663.1 |  |  |  | EU821860.1 |
|  |  |  |  |  | FJ238522.1 |  |  |  | EU821861.1 |
|  |  |  |  |  | FJ238523.1 |  |  |  | EU821867.1 |
|  |  |  |  |  | FJ238527.1 |  |  |  | EU339480.1 |
|  |  |  |  |  | FJ238529.1 |  |  |  | EU339481.1 |
|  |  |  |  |  | FJ238530.1 |  |  |  | EU339482.1 |
|  |  |  |  |  | FJ238531.1 |  |  |  | EU339483.1 |
|  |  |  |  |  | FJ358693.1 |  |  |  | FJ238525.1 |
|  |  |  |  |  | FJ358694.1 |  |  |  | EU673095.1 |
|  |  |  |  |  | FJ358695.1 |  |  |  | DQ093202.1 |
|  |  |  |  |  | FJ358696.1 |  |  |  | DQ356365.1 |
|  |  |  |  |  | FJ358697.1 |  |  |  | DQ356366.1 |
|  |  |  |  |  | FJ358698.1 |  |  |  | DQ008351.1 |
|  |  |  |  |  | EU673106.1 |  |  |  | DQ008352.1 |
|  |  |  |  |  | EF591951.1 |  |  |  | DQ008353.1 |
|  |  |  |  |  | EF591952.1 |  |  |  | DQ233632.1 |
|  |  |  |  |  | EF591953.1 |  |  |  | DQ233633.1 |
|  |  |  |  |  | EU137879.1 |  |  |  | AY615120.1 |
|  |  |  |  |  | EU137880.1 |  |  |  | AY615121.1 |
|  |  |  |  |  | DQ356360.1 |  |  |  | AY615144.1 |
|  |  |  |  |  | DQ008347.1 |  |  |  | AY615145.1 |
|  |  |  |  |  | DQ008348.1 |  |  |  | AY615168.1 |
|  |  |  |  |  | DQ008349.1 |  |  |  | AY615169.1 |
|  |  |  |  |  | DQ008350.1 |  |  |  | AY615170.1 |
|  |  |  |  |  | DQ233621.1 |  |  |  | AY615171.1 |
|  |  |  |  |  | DQ233622.1 |  |  |  | AY236909.1 |
|  |  |  |  |  | DQ233623.1 |  |  |  | AY236910.1 |
|  |  |  |  |  | DQ233624.1 |  |  |  | AY236911.1 |
|  |  |  |  |  | AY615178.1 |  |  |  | AY236912.1 |
|  |  |  |  |  | AY615179.1 |  |  |  | AY236913.1 |
|  |  |  |  |  | AY615180.1 |  |  |  | AY236914.1 |
|  |  |  |  |  | AY236924.1 |  |  |  | AY236915.1 |
|  |  |  |  |  | AY236925.1 |  |  |  | AY236916.1 |
|  |  |  |  |  | AY236926.1 |  |  |  | AY236917.1 |
|  |  |  |  |  | AY236927.1 |  |  |  | AY236918.1 |
|  |  |  |  |  | AY236928.1 |  |  |  | AY236919.1 |
