# Supplementary material for: High-Resolution Melting assays development for discrimination of fungal pathogens causing Grapevine Trunk Diseases
Source: PLoS One. 2025 Dec 1;20(12):e0331101. doi: 10.1371/journal.pone.0331101 (PMC12668526; doi:10.1371/journal.pone.0331101)
Supplement: S3 File — (DOCX) [file pone.0331101.s003.docx]

| Fungal species | Isolate | DNA concentration (ng/µL) | Absorbance ratio A_260_/A_280_ | Absorbance ratio A_260_/A_230_ |
| --- | --- | --- | --- | --- |
| *N. parvum* | 100.1 | 305.1 | 1.66 | 0.78 |
| *N. parvum* | 143.1 | 836.0 | 1.79 | 1.03 |
| *N. parvum* | 98.1 | 60.7 | 1.32 | 0.41 |
| *N. luteum* | 160.2 | 287.0 | 1.43 | 0.60 |
| *N. luteum* | 34.1 | 242.6 | 1.44 | 0.63 |
| *N. luteum* | 35.1 | 195.3 | 1.66 | 0.92 |
| *B. dothidea* | 94.1 | 84.6 | 1.51 | 0.61 |
| *B. dothidea* | 96.1 | 84.8 | 1.25 | 0.43 |
| *B. dothidea* | 100.3 | 388.2 | 1.75 | 0.91 |
| *Da. seriata* | 16.1 | 363.3 | 1.73 | 1.01 |
| *Da. seriata* | 21.5 | 307.1 | 1.75 | 0.99 |
| *Da. seriata* | 124.1 | 1616.8 | 1.46 | 0.96 |
| *Da. mutila* | 107.1 | 107.6 | 1.47 | 0.51 |
| *E. lata* | 98.3 | 1970.6 | 1.80 | 1.28 |
| *E. lata* | 46.3 | 1108.8 | 1.83 | 1.45 |
| *E. lata* | 49.8 | 531.1 | 1.62 | 0.90 |
| *Di. ampelina* | 39.1 | 104.7 | 1.61 | 0.68 |
| *Di. ampelina* | 153.1 | 509.4 | 1.41 | 0.95 |
| *Di. ampelina* | 67.2 | 485.3 | 1.50 | 0.79 |
| *Pa. chlamydospora* | 20.2 | 402.2 | 1.24 | 0.55 |
| *Pa. chlamydospora* | 206.7 | 1920.0 | 1.53 | 0.58 |
| *Pa. chlamydospora* | 20.1 | 527.9 | 1.28 | 0.77 |
| *Pm. minimum* | 66.1 | 272.9 | 1.79 | 0.85 |
| *Pm. minimum* | 66.2 | 301.8 | 1.74 | 0.84 |
| *Pm. minimum* | 293.2 | 385.1 | 1.81 | 1.11 |
| *F. mediterranea* | 295.1 | 526.6 | 1.74 | 1.15 |
| *F. mediterranea* | 36.2 | 1540.0 | 1.83 | 1.21 |
| *F. mediterranea* | 23.2 | 282.6 | 1.84 | 1.68 |
|  | Grapevine Sample | DNA concentration (ng/µL) | Absorbance ratio A_260_/A_280_ | Absorbance ratio A_260_/A_230_ |
|  | Grv-Bd | 23.0 | 1.56 | 0.59 |
|  | Grv-Bd/Ds | 15.9 | 1.43 | 0.32 |
|  | Grv-Ds | 52.9 | 1.46 | 0.38 |
